# Differentiating Sensitivity of Post-Stimulus Undershoot under Diffusion Weighting: Implication of Vascular and Neuronal Hierarchy

**DOI:** 10.1371/journal.pone.0002914

**Published:** 2008-08-13

**Authors:** Todd B. Harshbarger, Allen W. Song

**Affiliations:** Brain Imaging and Analysis Center, Duke University, Uninc Orange County, North Carolina, United States of America; University of Texas Southwestern Medical Center, United States of America

## Abstract

The widely used blood oxygenation level dependent (BOLD) signal during brain activation, as measured in typical fMRI methods, is composed of several distinct phases, the last of which, and perhaps the least understood, is the post-stimulus undershoot. Although this undershoot has been consistently observed, its hemodynamic and metabolic sources are still under debate, as evidences for sustained blood volume increases and metabolic activities have been presented. In order to help differentiate the origins of the undershoot from vascular and neuronal perspectives, we applied progressing diffusion weighting gradients to investigate the BOLD signals during visual stimulation. Three distinct regions were established and found to have fundamentally different properties in post-stimulus signal undershoot. The first region, with a small but focal spatial extent, shows a clear undershoot with decreasing magnitude under increasing diffusion weighting, which is inferred to represent intravascular signal from larger vessels with large apparent diffusion coefficients (ADC), or high mobility. The second region, with a large continuous spatial extent in which some surrounds the first region while some spreads beyond, also shows a clear undershoot but no change in undershoot amplitude with progressing diffusion weighting. This would indicate a source based on extravascular and small vessel signal with smaller ADC, or lower mobility. The third region shows no significant undershoot, and is largely confined to higher order visual areas. Given their intermediate ADC, it would likely include both large and small vessels. Thus the consistent observation of this third region would argue against a vascular origin but support a metabolic basis for the post-stimulus undershoot, and would appear to indicate a lack of sustained metabolic rate likely due to a lower oxygen metabolism in these higher visual areas. Our results are the first, to our knowledge, to suggest that the post-stimulus undershoots have a spatial dependence on the vascular and neuronal hierarchy, and that progressing flow-sensitized diffusion weighting can help delineate these dependences.

## Introduction

The typical functional magnetic resonance imaging (fMRI) study measures signal changes based on the blood oxygenation level dependent (BOLD) [1] effect. This BOLD signal consists of a number of characteristic components whose amplitudes and spatial extents may vary significantly [2], [3]. Specifically, a possible small initial decrease in signal is quickly followed by a large increase in signal, and at the cessation of the stimulus the signal decays over its baseline value and a signal undershoot is typically observed. The small initial dip in signal is not often seen without specialized and carefully formed methodology [4]. The main positive signal change is generally an increase on the order of 5%–8%, with a peak occurring approximately 5 seconds after the beginning of the stimulus [2], [3]. The post-stimulus undershoot signal typically peaks at about 5–10 seconds after the stimulus ends with a magnitude of approximately 1%–1.5% [5]–[7]. The stages of this BOLD signal result from the interplay of changes in cerebral blood flow (CBF), blood volume (CBV), and metabolism (CMRO_2_) related to activation of the underlying neurons. The normal measure of activation is based on the large positive increase, which is known to spread into the draining veins some distance from the site of neuronal activation.

Although consistently observed [5]–[7], the post-stimulus undershoot is not well understood and its implication on the fMRI signal localization can be unspecific given its omnipresence. The theories and experimental findings have insofar attributed the undershoot to one or more of three possible sources: the first is a delayed CBV recovery to baseline [8]–[10]; the second source is a sustained metabolic rate leading to increased oxygen extraction [11]–[15]; and the third potential source is a CBF undershoot [16]–[20]. These previous studies have investigated the temporal characteristics of the post-stimulus undershoot and its relation to CBF, CBV, or CMRO_2_. More recently, studies have indicated that the undershoot may be more specific to the site of neuronal activation than the large and diffuse positive BOLD response [21]. These high-resolution studies have shown that the undershoot originates in cortical layers with the highest metabolic activity. However, current research is still under debate as to its exact signal mechanism, for instance whether it is due to vascular or metabolic changes. Indeed, the high-resolution study recently reported [21] shows the undershoot spreading beyond the site of metabolic activity into other areas, indicating a dispersion of the signal which would not be specific to the neural activity. In addition, it is likely that the undershoot is not exclusively linked to one source specific to the neuronal activity. Other evidence has suggested that multiple sources may produce undershoot at differing spatial locations [11], [22], [23]. The variety of evidence related to the source of the post-stimulus undershoot indicates a complex relation between each of the three sources and their respective cortical localities. It would therefore be important to separate differing sources of the undershoot, in order to better characterize the underlying vascular or neuronal mechanisms.

In this current report, we hope to add evidence to better understand the BOLD undershoot by examining its dynamic response to increasing diffusion weighting sensitized to flow, and help better characterize its signal origin and mechanism. It has been shown that this flow-sensitive diffusion weighting strategy can be used to produce differentiation between signal arising from intravascular and extravascular sources, and further, from large vessels and capillaries [24]. However, a complete study of the effect of diffusion weighting on the post-stimulus undershoot has not been done. In a previous study, Jones [25] did not see a change in the undershoot with mild diffusion weighting, however, the level of diffusion weighting used was small. Obata, et al, [26] presented work showing an averaged change in the undershoot with high levels of diffusion weighting, but did not attempt to characterize the vasculature from which the signal arose. Here, we report on the possibility of separating intra- and extra- vascular sources of the post-stimulus undershoot based on their distinctly different behavior under diffusion weighting, and inferring their respective vascular and neuronal mechanisms. In particular, we expect that undershoot signal arising from intravascular sources will be affected by increasing diffusion weighting, whereas extravascular signal will not be attenuated. The spatial patterns of undershoot behavior should also be consistent with the separate vascular sources.

## Methods

A total of six healthy subjects were scanned for this study after providing written informed consent as approved by the Duke University Medical Center IRB. Scans were performed on a General Electric 4T whole-body MRI scanner (Waukesha, WI). For each study, high resolution anatomical images were first acquired for anatomical references, followed by a series of functional runs.

Functional scans were acquired using a gradient echo spiral imaging sequence with FOV = 24 cm, TE = 40 ms, a 128×128 imaging matrix, and a small flip angle of 30 degrees to reduce inflow effects. Eight slices (5 mm slice thickness, no gaps) aligned along the Calcarine fissure were selected covering the primary visual cortex. Three isotropic diffusion weighting b factors of 1, 63, and 125 s/mm^2^ were sequentially applied in series such that within a 3 sec repetition, volumes at three b factors were acquired [27], [28]. This interleaved acquisition approach reduces the effects of subject motion and other instabilities (e.g. scanner drifts) when comparing across the diffusion weighting strengths. Earlier studies have used diffusion weighting to suppress signals in larger vessels and gain improved functional localization [24], [29]–[31]. It has also been demonstrated that an appropriate range for the intra-voxel incoherent motion (IVIM) weighting [32], [33], especially by using an appropriate initial *b* factor to suppress arterial contributions, can be used to create an ADC contrast uniquely sensitive to the capillary networks [27], [28], [34], [35].

A total of 210 volumes (70 at each b-factor) were acquired while the subject observed a visual stimulus consisting of a flashing, rotating checkerboard. The visual stimuli were delivered using a goggle system (Resonance Technology, Northridge, CA). Subjects were instructed to maintain their gaze on a small fixation cross in the center of the visual field which was visible throughout the run. The stimulus alternated with a control condition of simply the fixation cross on a black screen. The rest and stimulation periods each lasted 30 seconds, and after an initial fixation period, the task and fixation pairs were repeated three times. This 30 second on/off paradigm is sufficient for our needs of detecting peak amplitudes of the undershoot, which occur soon after the cessation of the stimulus. Longer rest periods would be needed to characterize the full length and duration of the undershoot.

A total of six functional runs were acquired for each subject. The runs were averaged, and a multiple linear regression was performed on data from each b-factor separately to determine areas of activation. Three regressors were used: one based on the activation pattern, a second with a constantly increasing linear ramp function, and a third with a constant value. The maps corresponding to positive activation from each b-factor were examined and those voxels producing activation above a statistical threshold of z = 4 in all three maps were analyzed. Time courses of activation were determined from these active voxels, and linear drifts were removed for each individual voxel. The three stimulus blocks were averaged to determine a shortened time course. Since each b-factor is acquired at a slightly different position in time, the time courses for the different diffusion weightings were shifted to ensure temporal alignment. The initial fixation period provided a baseline to determine the percent change in signal, as well as a static measure of the apparent diffusion coefficient (ADC).

Active voxels were separated into three groups: those which showed undershoot and progressing amplitude reduction (reduction by >20%, which corresponds to signal reduction of free water) under diffusion weighting, those which showed undershoot but maintained constant amplitude, and those which did not show a significant undershoot at all. For individual voxels, the presence of an undershoot was determined by finding the mean of the signal 10 seconds following the cessation of the positive stimulus signal and comparing it to the baseline signal level. Those voxels in which the mean during the undershoot phase was not lower than the mean of the initial baseline signal were considered to have no undershoot. The mean was used rather than the peak magnitude so that noise spikes would not interfere with the determination of the undershoot. The reduction in undershoot magnitude under diffusion weighting was determined using the same signal means, and a separation value which based on our b-factors would separate large and small vessel contributions was used to divide voxels between the two groups with undershoot. Changes in this cut-off value or the b-factor values would alter the sensitivity of the technique to vessels of different blood mobility. The BOLD signal in the three groups was averaged first within subjects and across trials, then converted to percent change and averaged across subjects, The static ADC values were also determined for each group and averaged across subjects.

## Results

All subjects showed positive BOLD activation at each level of diffusion weighting. The extent of the activation was reduced by the amount of diffusion weighting, as described in our earlier work [28]. Given our moving visual stimuli, activation was seen throughout the visual cortex, extending from primary visual cortex (V1) into higher order visual areas (V4, MT). All of these areas were included in the analysis.

**Figure 1:**
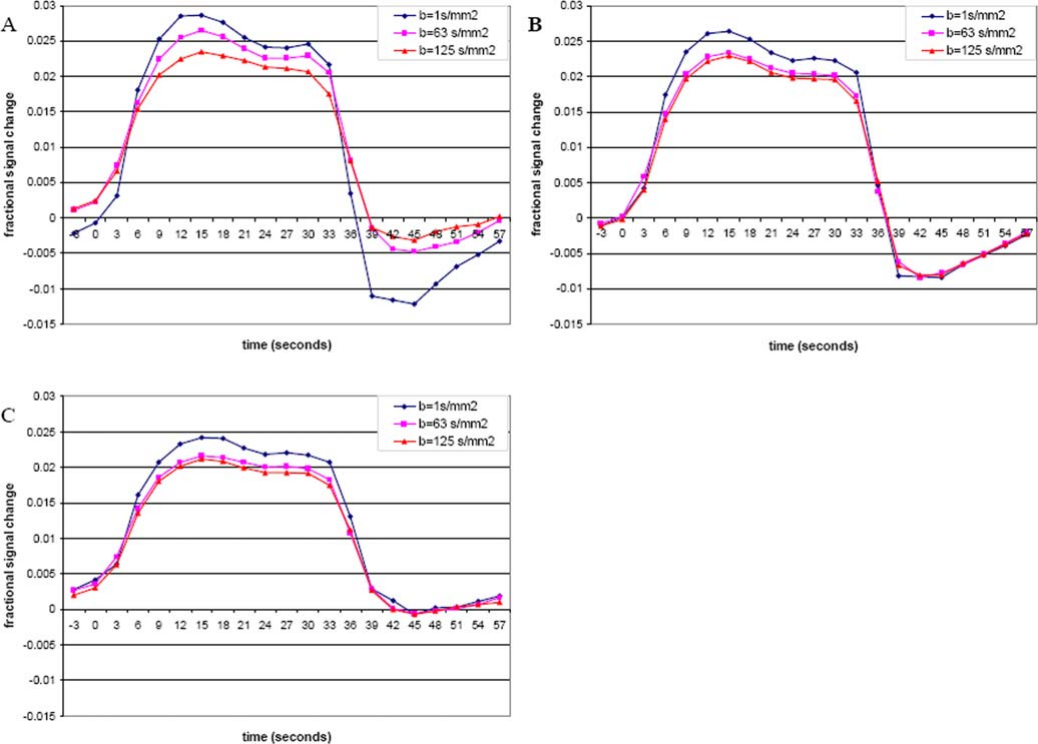
Time courses of activation for each of the three groups. A: Undershoot is observed to change with increasing diffusion weighting. B: Undershoot shows no change. C: No significant undershoot.

As described in the Methods, the active voxels were divided into three groups based on the distinctly different post-stimulus undershoot under progressing diffusion weighting–group 1 in which the undershoot was attenuated by diffusion weighting, group 2 in which the undershoot remained the same, and group 3 in which no significant undershoot was found. The average time courses for each of these groups are shown in Figure 1. The percent signal change shown here is based on the initial baseline value, and as such the initial value at the onset of activation in these average plots may be affected by the slow return to baseline of the undershoot and any variations that occur between stimulus periods. It can be seen that the change in positive signal level is approximately equal in each group, with group 3 having a slightly lower level of positive activation, although this did not reach significance at any level of diffusion weighting. For each group, the maximum positive signal was significantly greater (paired test across subjects, p<0.05) in the low diffusion weighting of b = 1 s/mm^2^ as compared to the two higher diffusion weightings of b = 63 s/mm^2^ and b = 125 s/mm^2^. The change in undershoot signal level, however, was distinctly different across all three levels of diffusion weighting for each group. Table 1 summarizes the maximum signals for both the positive response and undershoot for the three groups as appropriate, along with a ratio of undershoot to maximum signal.

**Table 1:** Signal comparisons.

	Undershoot Change (Average voxels = 267+/−66)			No undershoot change (1037+/−250 voxels)			No undershoot (2091+/−414 voxels)
	Positive BOLD	Undershoot	Ratio	Positive BOLD	Undershoot	Ratio	Positive BOLD
B = 1	2.64%	−1.43%	0.542	2.46%	−1.02%	0.415	2.20%
	+/−0.5%	+/−0.4%		+/−0.3%	+/−0.3%		+/−0.3%
b = 63	2.41%	−0.74%	0.307	2.15%	−1.05%	0.488	1.93%
	+/−0.3%	+/−0.5%		+/−0.2%	+/−0.5%		+/−0.4%
b = 125	2.15%	−0.56%	0.260	2.11%	−1.03%	0.488	1.86%
	+/−0.5%	+/−0.5%		+/−0.2%	+/−0.5%		+/−0.4%

Maximum percent changes in signal from baseline value (mean+/−standard deviation) for positive BOLD and post-stimulus undershoot, and ratio of magnitude of undershoot to positive peak. The average number of voxels (mean+/+/−standard deviation) across subjects are also indicated.

**Figure 2:**
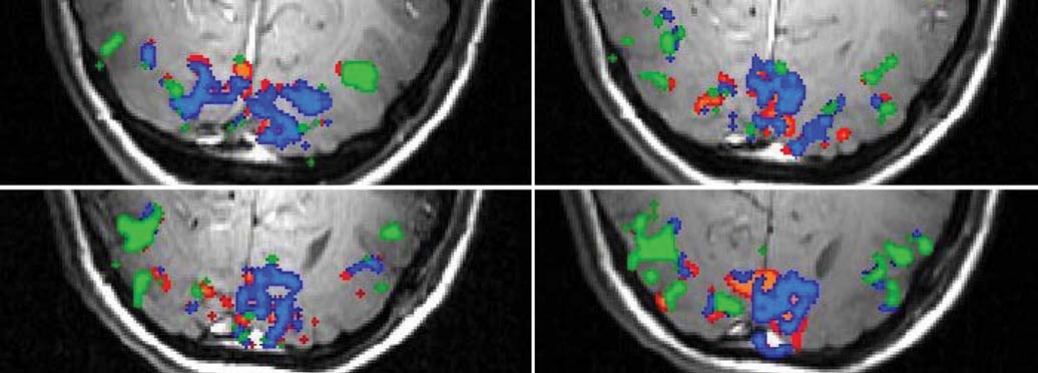
Activation overlays. Activation extent of the three groups determined by the undershoot response to diffusion weighting for a representative subject. Green areas represent areas showing no post-stimulus undershoot. Red areas represent those areas in which the undershoot was modified by the application of diffusion weighting. Blue areas represent regions in which the undershoot was unchanged.

**Figure 3:**
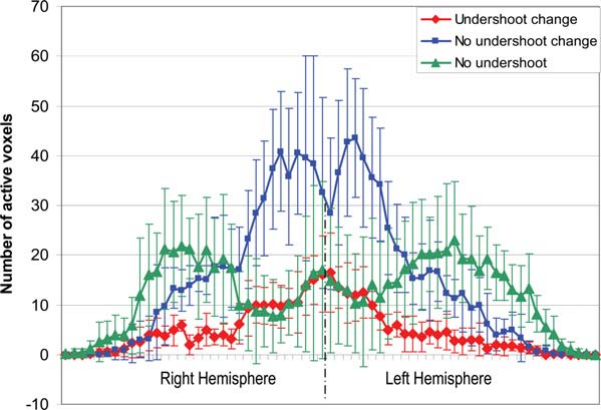
Activation distribution. Spatial distribution of the three regions from left to right, averaged across subjects. The error bars represent one standard deviation. The group showing no undershoot is clearly lateralized, while the two groups with observed undershoot show more centralized peaks.

The spatial extents of each of the three groups were also determined. Figure 2 shows the representative activation maps overlaid onto the high resolution anatomical images. Regions in group 1 are focal and have a limited spatial extent. Regions in group 2 immediately surround, but also extend well beyond, the vicinity of group 1. Areas in group 3 were largely in higher order visual areas lateral and anterior to the primary visual cortex. This pattern was consistently observed in all subjects, and the overall number of active voxels across subjects was similar. To allow a comprehensive assessment of the spatial distribution of these groups, a composite spatial histogram (projections from left to right of the brain) including all activated voxels in all subjects was obtained, as shown in Figure 3. Specifically, for each subject, the number of voxels were collapsed onto the horizontal axis, and then aligned by centering the interhemispheric fissure and averaged across all subjects.

While spatial continuation was observed within each group, a clear separation of these groups was also evident suggesting their individual spatial sensitivities. In particular, the voxels showing post-stimulus undershoot are largely distributed near the primary visual cortex, and the voxels with no undershoot are largely grouped towards the high visual areas. More specifically, the voxels in group 1 made up 21% of active voxels showing an undershoot and 8% of active voxels overall. Group 2 comprised 79% and 30% of active voxels with undershoot and overall, respectively. Group 3, made up of voxels showing no significant undershoot, covered the largest area and made up 62% of all active voxels. The majority of voxels in this third group were located in higher visual areas.

The static ADC values were also determined for each of these groups. The average ADC in the group exhibiting a significant change in undershoot was the highest, with a value of 1.75×10^−3^ (+/−0.37) mm^2^/s. The group which had undershoot, but did not experience a change under diffusion weighting showed an average ADC of 1.46×10^−3^ (+/−0.17) mm^2^/s. Finally, the group with no undershoot was observed to have an ADC value between the other two groups, at 1.55×10^−3^ (+/−0.45) mm^2^/s. While significant partial volume effect remains, which may give rise to an averaged ADC of the various groups within any given voxel, the clear separation of the ADC values does provide insight on the dominant signal source within each group.

## Discussion

### Conclusions

Using flow-sensitized diffusion weighting, we were able to separate the BOLD response into three distinct groupings based on the characteristics of the post-stimulus undershoot. These groups show separate spatial distributions with large differences in extent and locality. Diffusion weighting will reduce the signal from intravascular spins, and has been shown to reduce the positive BOLD signal peak [24], [30]. Likewise, undershoot signal arising from intravascular spins in large vessels would also be reduced by the application of diffusion weighting. Although the activated voxels presented in this report all showed some reduction in positive signal (with minor differences in signal magnitude changes), voxels with signal reduction in the undershoot as well are limited to a small, localized, spatial extent and likely represent larger veins. This is further confirmed by the higher ADC values displayed within this group. The similarity in the response of the positive BOLD signal in the presence of undershoot disparities underscores the lack of specificity in the positive BOLD signal, and the potential for greater localization when the undershoot is also considered in the analysis.

A second group of voxels exhibited undershoot; however, its amplitude was not attenuated by the presence of diffusion weighting. Since the extravascular component of the large veins as well as a capillary signal source will not show significant signal reduction under mild diffusion weighting, given their mostly parenchyma origins, it is likely that the sources of this second group would be within these proton pools. In our data, this group is seen to have a larger spatial extent, some surrounding the larger veins identified in the first group and the rest remaining distal from the large veins but spatially continuous. This observation is consistent with these two potentially contributing sources. The lower average ADC values also suggest extravascular and small vessel origins. The small attenuation seen in the positive BOLD activation within this area is likely from the limited intravascular contribution of the small vessels, which may include venules and capillaries.

The lack of any undershoot in the third group could have a number of different causes. The type and duration of a stimulus [18], [36], and thus metabolic demand, can affect whether or not undershoots are observed. In addition, regional differences in the regulation of perfusion or vasculature or other anatomic differences [23], [37], [38] can affect the BOLD signal characteristics. In particular, since the BOLD signal is based on vascular responses, the signal will depend on the density of fine vascularization and capillary beds [39]. Marcar, et al, [40] found that the higher vascular density in area V1, as opposed to extra-striate cortex, led to differences in the responses of these areas to stimuli, a prediction made earlier by Zheng, et al, [41]. In addition, Huettel, et al, [42] found that the relation between ERP's and BOLD response differed between brain regions, possibly related to the local vasculature. The ratio of change in CBF to CMRO2 has been shown to vary between brain regions [43], [44], indicating that the BOLD response does not represent neuronal activity identically throughout the brain. Outside of visual areas, Obata, et al, [45] also showed different BOLD responses in primary motor cortex versus supplemental motor areas (SMA), with a much smaller undershoot within the SMA. Chiarelli, et al, [44] also showed a much greater undershoot within visual cortex than for motor cortex. Smaller scale differences between surface and tissue vessels will also affect the undershoot signal. Yacoub, et al, [22] found that different physiological mechanisms seemingly control the stimulus undershoot for surface and tissue vessels. Specifically, they found undershoot in both regions, despite differing CBV responses. Zhao, et al, [21] found a similar difference in CBV, positive BOLD and undershoot across the cortical layers. These results may indicate that the mismatch between CBV, CBF, and CMRO_2_ that can produce a signal undershoot may differ across both cortical depth as well as larger functionally distinct brain areas. Using PET methodology, Ishii, et al, [46] showed regional differences in resting state metabolism, CBF, CBV, and oxygen extraction. These differences in baseline conditions will affect the task-related response, as well.

In our experiments, the distinct localization of group 3 in higher order visual areas is consistently observed across subjects, as shown in a composite spatial histogram in Fig. 3. If the undershoot is primarily a vascular phenomenon, then it should also exist in higher visual areas where both large and small vessels are present. Thus the consistent observation of this group would argue against a vascular origin and support a metabolic basis for the post-stimulus undershoot. Specifically, the undershoot may be due to a sustained oxygen metabolism after the cessation of brain functions [47], [48], potentially to restore ion concentrations across the neuronal membrane [49] and/or restore ATP reserves that were consumed by the metabolic demand in regions with high brain activation incommensurate to the energy supply. It could then be inferred that the lack of an undershoot within the high-order visual areas is possibly due to a lower metabolic demand in higher visual regions, which are relayed by the secondary areas from the primary visual cortex during visual stimulation. Further, the differentiation of regions based on the amplitude of the post-stimulus undershoot may provide a new possibility to infer functional hierarchy of the active brain networks among activated regions. To fully realize such a classification, work is still needed to accurately determine the source of the undershoot and any variations which may exist across brain regions.

In conclusion, our study provides further evidence on the mechanisms of post-stimulus undershoot. If the primary cause of the undershoot is a general increase in large veins [8] after the cessation of the neuronal stimulation, then only the first group, which showed signal changes with diffusion weighting, and a subset of the second group immediately surrounding the first group will be observed in our experiments. However, the spatially diffuse, yet continuous pattern of the second group often distal from the large veins shown in our experiments suggests that there is a significant source for the undershoot other than the large veins. It is likely that a sustained high oxygen metabolism originated from the small vessels may contribute to this phenomenon. Furthermore, the emergence of the third group in higher visual areas, in which no undershoot was detected, would suggest the lack of sustained metabolic demand after activation, as the result of a lower metabolic demand during visual stimulation. These findings suggest that our method may be sufficiently sensitive to differentiate the various activated regions within an active brain network, and with further confirmation of the undershoot source, may allow for the inference of vascular and nueronal heirarchy within those networks.

## References

[1] Ogawa S, Lee TM, Kay AR, Tank DW (1990). Brain magnetic resonance imaging with contrast dependent on blood oxygenation.. Proc Nat Acad Sci U S A.

[2] Buxton RB, Uludag K, Dubowitz DJ, Liu TT (2004). Modeling the hemodynamic response to brain activation.. Neuroimage.

[3] Fransson P, Kruger G, Merboldt KD, Frahm J (1998). Temporal characteristics of oxygenation-sensitive MRI responses to visual activation in humans.. Magn Reson Med.

[4] Buxton RB (2001). The elusive initial dip.[comment].. Neuroimage.

[5] Chen W, Zhu XH, Kato T, Andersen P, Ugurbil K (1998). Spatial and temporal differentiation of fMRI BOLD response in primary visual cortex of human brain during sustained visual simulation.. Magn Reson Med.

[6] Frahm J, Kruger G, Merboldt KD, Kleinschmidt A (1996). Dynamic uncoupling and recoupling of perfusion and oxidative metabolism during focal brain activation in man.. Magn Reson Med.

[7] Kruger G, Kleinschmidt A, Frahm J (1996). Dynamic MRI sensitized to cerebral blood oxygenation and flow during sustained activation of human visual cortex.. Magn Reson Med.

[8] Buxton RB, Wong EC, Frank LR (1998). Dynamics of blood flow and oxygenation changes during brain activation: the balloon model.. Magn Reson Med.

[9] Jones RA, Schirmer T, Lipinski B, Elbel GK, Auer DP (1998). Signal undershoots following visual stimulation: a comparison of gradient and spin-echo BOLD sequences.. Magn Reson Med.

[10] Mandeville JB, Marota JJ, Ayata C, Zaharchuk G, Moskowitz MA (1999). Evidence of a cerebrovascular postarteriole windkessel with delayed compliance.. J Cereb Blood Flow Metab.

[11] Jin T, Wang J, Zhao F, Wang P, Tasker M (2006). Spatiotemporal characteristics of BOLD, CBV, and CBF responses in the cat visual cortex.. 14th Annual Meeting of the ISMRM, Seattle, Washington.

[12] Lu H, Golay X, Pekar JJ, Van Zijl PC (2004). Sustained poststimulus elevation in cerebral oxygen utilization after vascular recovery.. J Cereb Blood Flow Metab.

[13] Poser BA, Norris DG (2007). On bursting balloons and collapsing endothelia: What does cause the post-stimulus undershoot?. 15th Annual Meeting of the ISMRM, Berlin, Germany.

[14] Schroeter ML, Kupka T, Mildner T, Uludag K, von Cramon DY (2006). Investigating the post-stimulus undershoot of the BOLD signal–a simultaneous fMRI and fNIRS study.. Neuroimage.

[15] Tuunanen PI, Vidyasagar R, Kauppinen RA (2006). Effects of mild hypoxic hypoxia on poststimulus undershoot of blood-oxygenation-level-dependent fMRI signal in the human visual cortex.. Magn Reson Imag.

[16] Behzadi Y, Restom K, Perthen J, Liu TT (2006). Reducing inter-voxel variability of the BOLD response with measurement of resting blood flow.. 14th Annual Meeting of the ISMRM, Seattle, Washington.

[17] Chen JJ, Advani K, Pike GB (2007). Characterization of the BOLD Post-Stimulus Undershoot.. 15th Annual Meeting of the ISMRM, Berlin, Germany.

[18] Hoge RD, Atkinson J, Gill B, Crelier GR, Marrett S (1999). Stimulus-dependent BOLD and perfusion dynamics in human V1.. Neuroimage.

[19] Uludag K, Dubowitz DJ, Yoder EJ, Restom K, Liu TT (2004). Coupling of cerebral blood flow and oxygen consumption during physiological activation and deactivation measured with fMRI.. Neuroimage.

[20] Friston KJ, Mechelli A, Turner R, Price CJ (2000). Nonlinear responses in fMRI: the Balloon model, Volterra kernels, and other hemodynamics.. Neuroimage.

[21] Zhao F, Jin T, Wang P, Kim SG (2007). Improved spatial localization of post-stimulus BOLD undershoot relative to positive BOLD.[see comment].. Neuroimage.

[22] Yacoub E, Ugurbil K, Harel N (2006). The spatial dependence of the poststimulus undershoot as revealed by high-resolution BOLD- and CBV-weighted fMRI.. J Cereb Blood Flow Metab.

[23] Zhu X-H, Zhang Y, Zhang N, Park J, Chen W (2006). Study of Post-stimulus BOLD Undershoots between Lateral Geniculate Nucleus and Primary Visual Cortex in Cat Brain.. 14th Annual Meeting of the ISMRM, Seattle, Washington.

[24] Song AW, Wong EC, Tan SG, Hyde JS (1996). Diffusion weighted fMRI at 1.5 T.. Magn Reson Med.

[25] Jones RA (1999). Origin of the signal undershoot in BOLD studies of the visual cortex.. NMR in Biomed.

[26] Obata T, Tomiyasu M, Kashikura K, Hirano Y, Nonaka H (2006). Minimization of Post-Stimulus Undershoot in Heavily Diffusion-Weighted Functional Magnetic Resonance Imaging.. 14th Annual Meeting of the ISMRM, Seattle, Washington.

[27] Gangstead SL, Song AW (2002). On the timing characteristics of the apparent diffusion coefficient contrast in fMRI.. Magn Reson Med.

[28] Song AW, Woldorff MG, Gangstead S, Mangun GR, McCarthy G (2002). Enhanced spatial localization of neuronal activation using simultaneous apparent-diffusion-coefficient and blood-oxygenation functional magnetic resonance imaging.. Neuroimage.

[29] Boxerman JL, Bandettini PA, Kwong KK, Baker JR, Davis TL (1995). The intravascular contribution to fMRI signal change: Monte Carlo modeling and diffusion-weighted studies in vivo.. Magn Reson Med.

[30] Michelich CR, Song AW, Macfall JR (2006). Dependence of gradient-echo and spin-echo BOLD fMRI at 4 T on diffusion weighting.. NMR in Biomed.

[31] Zhong J, Kennan RP, Fulbright RK, Gore JC (1998). Quantification of intravascular and extravascular contributions to BOLD effects induced by alteration in oxygenation or intravascular contrast agents.. Magn Reson Med.

[32] Le Bihan D, Breton E, Lallemand D, Grenier P, Cabanis E (1986). MR imaging of intravoxel incoherent motions: application to diffusion and perfusion in neurologic disorders.. Radiology.

[33] Le Bihan D, Turner R (1992). The capillary network: a link between IVIM and classical perfusion.. Magn Reson Med.

[34] Darquie A, Poline JB, Poupon C, Saint-Jalmes H, Le Bihan D (2001). Transient decrease in water diffusion observed in human occipital cortex during visual stimulation.. Proc Nat Acad Sci U S A.

[35] Harshbarger TB, Song AW (2004). B factor dependence of the temporal characteristics of brain activation using dynamic apparent diffusion coefficient contrast.. Magn Reson Med.

[36] Bandettini PA, Kwong KK, Davis TL, Tootell RB, Wong EC (1997). Characterization of cerebral blood oxygenation and flow changes during prolonged brain activation.. Human Brain Mapping.

[37] Kamba M, Sung Y-W, Ogawa S (2007). Alteration of Blood Oxygenation Level-Dependent Signaling by Local Circulatory Condition.. J Magn Reson Imag.

[38] Wolf M, Wolf U, Toronov V, Michalos A, Paunescu LA (2002). Different time evolution of oxyhemoglobin and deoxyhemoglobin concentration changes in the visual and motor cortices during functional stimulation: a near-infrared spectroscopy study.. Neuroimage.

[39] Harrison RV, Harel N, Panesar J, Mount RJ (2002). Blood capillary distribution correlates with hemodynamic-based functional imaging in cerebral cortex.[see comment].. Cerebral Cortex.

[40] Marcar VL, Loenneker T, Straessle A, Girard F, Martin E (2004). How much luxury is there in ‘luxury perfusion’? An analysis of the BOLD response in the visual areas V1 and V2.. Magn Reson Imag.

[41] Zheng D, LaMantia AS, Purves D (1991). Specialized vascularization of the primate visual cortex.. J Neurosci.

[42] Huettel SA, McKeown MJ, Song AW, Hart S, Spencer DD (2004). Linking hemodynamic and electrophysiological measures of brain activity: evidence from functional MRI and intracranial field potentials.. Cerebral Cortex.

[43] Ances BM, Leontiev O, Perthen JE, Liang C, Lansing AE (2008). Regional differences in the coupling of cerebral blood flow and oxygen metabolism changes in response to activation: Implications for BOLD-fMRI.. NeuroImage.

[44] Chiarelli PA, Bulte DP, Gallichan D, Piechnik SK, Wise R (2007). Flow-metabolism coupling in human visual, motor, and supplementary motor areas assessed by magnetic resonance imaging.. Magn Reson Med.

[45] Obata T, Liu TT, Miller KL, Luh WM, Wong EC (2004). Discrepancies between BOLD and flow dynamics in primary and supplementary motor areas: application of the balloon model to the interpretation of BOLD transients.. Neuroimage.

[46] Ishii K, Sasaki M, Kitagaki H, Sakamoto S, Yamaji S (1996). Regional difference in cerebral blood flow and oxidative metabolism in human cortex.. J Nucl Med.

[47] Aubert A, Pellerin L, Magistretti PJ, Costalat R (2007). A coherent neurobiological framework for functional neuroimaging provided by a model integrating compartmentalized energy metabolism.. Proc Nat Acad Sci U S A.

[48] Kasischke KA, Vishwasrao HD, Fisher PJ, Zipfel WR, Webb WW (2004). Neural activity triggers neuronal oxidative metabolism followed by astrocytic glycolysis.[see comment].. Science.

[49] Attwell D, Iadecola C (2002). The neural basis of functional brain imaging signals.. Trends Neurosci.

